# Factors that Influence Chinese Parents’ Intentions to Use Physical Violence to Discipline Their Preschool Children

**DOI:** 10.3390/ijerph17051787

**Published:** 2020-03-10

**Authors:** Haixue Wang, Guangrong Zhu, Jingqi Chen, Linjing Lyu, Michael Dunne

**Affiliations:** 1 Institute of Child and Adolescent Health, School of Public Health, Peking University, Beijing 100191, China; gwwanghaixue@126.com (H.W.); zhuguangrong@bjmu.edu.cn (G.Z.); LLJ1160550662@163.com (L.L.); 2 School of Public Health and Social Work, Queensland University of Technology, Brisbane 4000, Australia; m.dunne@qut.edu.au; 3 Institute for Community Health Research, Hue University, Hue 530000, Vietnam

**Keywords:** physical violence against children, parents, intentions, theory of planned behavior, China

## Abstract

This study explored factors affecting parents’ intentions to use physical violence (PV) to discipline their children in the future. The theory of planned behavior (TPB) guided selection of variables. A sample of 1337 preschool children’s parents from nine kindergartens located in a county of Henan Province, China were selected by stratified random cluster sampling. Data on parents’ attitudes, subjective norms, and perceived behavioral control over PV, intentions to engage in PV to discipline their preschool children in the future, self-reported PV behavior toward their children during the past three months, and demographic characteristics were collected via a paper-based questionnaire. Multivariable logistic regression analyses examined putative predictors of parents’ intentions to use physically violent discipline. Nearly three-quarters of the sample said they definitely will not use violent discipline, while 23.4% either said they would use it, or did not rule it out. Logistic regression analysis showed that parents’ lower level of perceived behavioral control over using violence (*OR* 4.17; 95% *CI*: 2.659, 6.551), attitudes that support PV (*OR* 2.23; 95% *CI*: 1.555, 3.203), and having been physically violent with their children during the past three months (*OR* 1.62; 95% *CI*: 1.032, 2.556) were significantly associated with parents’ tendency either to include, or not exclude, the use of violent discipline. Parents’ subjective norms regarding PV had no significant impact on their intentions (*p* > 0.05). The influence of TPB constructs varied according to parents’ gender. Intervention programs that aim to reduce violent discipline should focus both on increasing parents’ perceived behavioral control over PV and changing their attitudes toward physically violent practices, especially among mothers and parents who have already used PV to discipline their children.

## 1. Introduction

Physical violence (PV) against children has been increasingly reported as an important risk factor for impaired psychological development [1,2]. When examined with other adversities, childhood physical violence contributes independently to depression and anxiety throughout life [1]. Young people exposed to physical abuse may be 50.0% more likely to suffer depressive disorders than their non-abused peers [3]. Spanking experienced during childhood is linked with development of mental health problems among adults, such as suicide attempts, moderate to heavy drinking, the use of street drugs [4] and depression during old age [5]. Further, childhood physical maltreatment can lead to low educational attainment, unemployment, low earnings, and fewer material assets acquired during adulthood [6,7].

However, despite the weight of evidence that physical violence against children often precedes mental, physical, and social problems [8,9,10,11], it is still highly prevalent worldwide [12]. For example, a meta-analysis showed that the lifetime prevalence of any form of PV against children in China was about 36.6% [13]. Generally, children aged 2–8 years are most vulnerable to PV [14,15], and this higher risk may in part be due to their developmental immaturity and associated inability to comprehend and articulate the violence [16]. Research with preschool children in mainland China has shown estimates of the prevalence of parental PV behavior against preschool children range from 37.4% to 77.7% [17,18,19].

Parents are the main perpetrators of child PV [20,21] and their use of discipline strategies is generally based on their personal beliefs and is shaped by their experiences and cultural factors [22]. Chinese parents who experienced harsh discipline from their parents appear more likely to use violent discipline with their children [23]. Under the influence of traditional concepts that “beating is caring and scolding is loving” and “spare the rod and spoil the child” [24], many parents in China still consider that educating or disciplining children through beating, emotional abuse or other forms of violence is legitimate and effective [25].

Such beliefs and behavior are common but definitely not universal. Demographic characteristics explain some variation in the likelihood of PV. Parents’ gender, age, educational level, income, children’s age, sex, disability status, and school performance [26] may all be related to risk of PV as a disciplinary practice. In addition, parents’ attitudes that support use of corporal punishment shape parents’ PV behavior [23]. Mothers who believe that PV can lead to positive outcomes, including immediate compliance and long-term good behavior and better educational achievement use violence more frequently than mothers who do not share these beliefs [27].

One limitation of research in this field is that many studies examining determinants of PV are atheoretical. Much research is simply observational, which limits a systematic examination of factors that may drive harmful parental behavior. According to the theory of planned behavior (TPB) [28], an individual’s actual behavior can be predicted by their intentions to perform the target behavior: the stronger of the intentions, the higher probability that the behavior will happen. TPB emphasizes the role of attitudes and perceived social norms regarding a behavior in shaping behavioral intentions, and also recognizes the importance of perceived behavioral control in predicting behavioral intentions and actions. TPB is very useful in understanding intentions in situations where the behavior is not completely under the actor’s volitional control. This theoretical approach is suitable for PV because harsh, controlling parenting often escalates to severe emotional and physical maltreatment [29].

TPB has been widely applied in health psychology [30], including studies of violent behavior toward children. For instance, research into intergenerational transmission of discipline strategies [31] has explored pre-parents’ (e.g., college students) discipline intentions, and suggested reducing negative parenting styles in pre-parent populations [32,33]. In addition, some literature has focused on nurses’ and teachers’ intentions to report child violence behaviors, and emphasized the importance of conducting training programs on child abuse among these groups [16,34,35]. Those studies have empirically supported the application of TPB to aspects of child violence prevention.

Currently, although many studies have focused on factors affecting parents’ PV behavior, research specifically examining parents’ intentions to use PV against their preschool children, and its related influencing factors, is limited, especially within the theoretical framework of TPB. In this study, we examine whether TPB provides a useful approach for explaining parents’ intentions to engage in PV against their preschool children in China.

## 2. Methods

### 2.1. Study Population

This paper is derived from a cross-sectional survey conducted in a county of Zhumadian City, Henan Province, China [36]. Nine kindergartens, including 4 from the urban areas and 5 from rural areas were selected by using stratified random cluster sampling. In pre-schools where the number of children was less than or equal to 300, all children were selected for the sample. If the pre-school had many more than 300 children, then three classes in each grade were selected. Among them, 2121 enrolled children’s parents (father or mother) were invited to take part in the study, and 1337 children’s parents participated, accounting for 63.0% of registered students.

### 2.2. Measures

#### 2.2.1. Key Outcome Variable: Parents’ Intentions to Use PV toward Their Preschool Children in the Future

Parents’ intentions to use PV to discipline their children was measured by one item: “When your children make mistakes/misbehave, would you like to discipline your child by violent ways in the future (e.g., hitting, scolding, etc.)?” Responses were rated on a 5-point Likert including strongly not intend to, not intend to, not sure, intend to, and strongly intend to. Parents who answered “strongly not intend to” or “not intend to” were classified as not intending to use PV in the future, and parents who answered “strongly intend to” or “intend to” was defined as intend to use PV in the future. In this study, to better understand reasons why some parents did not definitely state they do not intend to use PV in the future, we classified parents’ intentions to use PV into two categories, that is (1) parents who definitely answered that they do not intend to use PV in the future and (2) parents who declined to state definitely that they will not use PV in the future (parents who answered that they intend to use PV and those who said they are not sure whether they will use PV in the future).

#### 2.2.2. Key Independent Variables: Constructs of TPB

##### Parents’ Attitudes toward Corporal Punishment to Discipline Children

The attitudes scale has been used in previous studies [23,37].The scale includes 5 items where parents were asked whether they approve the following statements: (1) In order to ensure children follow the rules, it is necessary to hit; (2) In order to enable children to discriminate between good and bad, it is necessary to hit them; (3) Spare the rod, spoil the child; (4) Parents can hit their children when their child is talking back or crying; (5) It is family affair whether parents hit and scold their children, and outsiders should not intervene. Item responses are on a five-point Likert scale including strongly disapprove, disapprove, not sure, approve, strongly approve. Parents considered to have positive attitudes toward non-violent discipline were those who disapproved or strongly disapproved of all statements in this scale. Otherwise, parents who approved or strongly approved or were not sure of one or more items were considered to have attitudes that may support PV. The Cronbach’s α of the scale was 0.81 [36].

##### Parents’ Subjective Norms regarding Physical Violence

This scale was self-designed and included 5 items. Participants were asked about their perceptions of other adults’ PV behavior to their children in the local community. The items in the scale are as follows: (1) Most people who are important to me use physical violence (like spanking) as a routine way to manage their children; (2) Most people who are close to me think that I should hit my child when he or she misbehaves; (3) People around me expect me to spank my child with my hand/object when he or she misbehaves; (4) Most parents like me hit their children when their children misbehave; (5) People in my life whose opinion are important to me hit their children when their children misbehave. Responses are on a five-point Likert scale including strongly approve, approve, neutral, disapprove, strongly disapprove, which were coded from 1 to 5 points, respectively. A composite score was obtained by summing responses to items with higher scores reflecting higher levels of subjective norms regarding physical violence of parents. Scores ranged from 5–25, and two levels of subjective norms were divided based on the score. Scores of 5–19 were classified as moderate/low level and 20–25 were classified as high level. The Cronbach’s α of the scale was 0.85.

##### Parents’ Perceived Behavioral Control over Physical Violence

This scale was self-designed and included 6 items. Participants were asked about the difficulty they perceived when they use non-violent discipline to educate their children. The items in the scale are as follows: (1) When my child makes a mistake/misbehaves, I hit he/she because I can’t control myself; (2) When my child makes a mistake/misbehaves, it’s up to me to hit him/her or not; (3) Our family members have different ways of disciplining children. For example, when a child makes a mistake, some people intend to discipline the child by hitting or scolding, while others don’t intend to; (4) When my child makes a mistake/misbehaves, I can’t think of a better way to discipline him other than by hitting him/her; (5) I will hit my child when he/she makes a mistake/misbehaves; (6) I feel that it is difficult for me to use non-violent discipline when my child makes a mistake/misbehaves. Responses were rated on a five-point Likert scale from always to never (from item 1 to item 4), very possible to very impossible (item 5), and strongly approve to strongly disapprove (item 6). The answers of each item were coded from 1 to 5 points, respectively. These items were summed to form a composite score of perceived behavioral control, the score range was 6–30, and two levels of perceived behavioral control were divided based on the score. The score of 6–24 was defined as moderate/low level and 25–30 was defined as high level. The Cronbach’s α of the scale was 0.85 in this study.

#### 2.2.3. Covariates

##### Parents’ Physical Violence Behavior against Children during the Past Three Months

The PV scale was adopted from a study conducted in primary school children’s parents [23], and has been used with parents of children with hearing loss [37]. It was developed based on items used in prior research [18,19,38,39,40,41]. The scale included 8 items as follows: (1) Pushed or shook a child; (2) pinched or scratched a child; (3) hit child’s buttocks with hand; (4) hit child’s hand, foot, back, arm or leg with hand; (5) hit child’s face or head with hand; (6) hit child’s buttocks with an object; (7) hit elsewhere (not buttocks) with an object; and (8) kicked a child with a foot or hit with a fist. For each item, parents were asked how often they did these acts with their children during the past three months. The response categories included “never”, “1–2 times”,”3–5 times”, “6–10 times”, and “more than 10 times”. PV was defined as having one or more behaviors among the eight items during the past three months. The Cronbach’s α reported in the previous study with the elementary school pupils’ parents and with hearing loss children’s parents were 0.85 [23] and 0.79 [37], respectively, and it was 0.85 in the present study [36].

For more information about the reliability and validity of these scales (the three TPB construct scales and the PV scale) see the Supplementary Materials (Tables S1–S4). The values of various fit indices indicated that these measurement models had a satisfactory fit.

##### Demographic Characteristics

Parents were asked to provide information regarding their gender, age, educational attainment (junior high school or less/senior high school or higher), marital satisfaction (5-point Likert scale, from very satisfied to very unsatisfied), family economic situation in the local area (5-point Likert scale, from very rich to very poor), and childhood experiences of PV victimization before 16 years old. Parents’ childhood experiences of PV victimization before 16 years old were measured by 2 items, including “Did your parents hit you with hand before you were 16 years old?” and “Did your parents hit you with an object before you were 16 years old?”. They were also asked to report the characteristics of their children, including child age, gender and only-one child or not.

This study was approved by Peking University Institutional Review Board (IRB00001052-18014). Anonymous questionnaire was used to protect respondent privacy. Parents were informed in the front of the questionnaire that participation is voluntary, and they could withdraw from the survey at any time. An envelope containing the questionnaire was distributed to parents when they picked up their children after school, and the completed questionnaire was sealed in an envelope by each parent before returning it to the research team. Parents who filled in the questionnaire and returned them to the researcher were considered to have agreed to participate in the survey. The survey was designed and administered using Chinese language. Before the present survey, a pilot study was conducted to demonstrate feasibility and gage parents’ willingness to complete all sections.

### 2.3. Data Analysis

Data were analyzed by SPSS software (version 20.0; SPSS Inc., Chicago, IL, USA). The Pearson chi-square test was used to examine differences of the intention rate among different subgroups of the sample. Three multivariate logistic regression models were employed to examine associations between TPB constructs and parents’ intentions to use PV in the future. The first model examined the independent effects of TPB constructs on parental intentions to use PV in the future, the second model examined the simultaneous effects of TPB constructs and parents’ PV behavior toward their children during the past three months on parental intentions to use PV in the future, and the third model explored the simultaneous effects of TPB constructs and parents’ PV behavior toward their children during the past three months, controlling for demographic characteristics. In the multivariate logistic regression models, the group of parents who definitely will not use violent discipline in the future were taken as the reference category.

## 3. Results

Respondents’ demographic characteristics are shown in Table 1. Of the 1337 parents, the mean age was 32.4 years, and about half reported that they did not complete 12 years of high school. Nearly 68.0% of parents admitted some acts of PV behavior against their preschool children in the past three months. The percent of parents who answered that they definitely intend to use PV in the future is 2.1%, while 21.3% said they were not sure whether they will use or not use PV in the future. In total, 23.4% of parents declined to state definitely that they will not use PV in the future. The intentions of parents’ to use PV varied according to urban-rural area, parents’ age, educational level, marital satisfaction, parental PV experience before 16, and whether or not they had a one child family.

Descriptive statistics for constructs of TPB and bi-variable associations with parents’ intentions to use PV in the future are shown in Table 2. The proportion of parents who declined to state definitely that they will not use PV in the future was higher among parents with attitudes supportive of PV (32.8%), among parents with moderate/low level of subjective norms (30.6%) and among those who perceived less behavioral control (33.9%) over physical violence than their counterparts (12.8%, 15.3%, and 7.4%, respectively). The bi-variable analysis indicates that all constructs of TPB, including parents’ attitudes, subjective norms, and perceived behavioral control over the use of corporal punishment to discipline children were associated with parents’ intentions to use PV in the future (*p* < 0.001). Besides, parents who already had used PV on their children during the past three months also had higher intention to use PV or were not sure about it, in the future (*p* < 0.001).

The results of logistic regression analyses are shown in Table 3. Three models were conducted to fully explore the effect of TPB variables on parents’ intentions to use PV toward their children. For all three models we can consistently see that, except for subjective norms, parents’ attitudes and perceived behavioral control over PV were significant factors associated with parents’ intentions of using PV to discipline their preschool children. Compared with attitudes, perceived behavioral control had higher influence in shaping parents’ intentions to use PV. For example, in the full model (Model 3), parents with moderate/low perceived behavioral control over PV had 4.17 times (95% *CI*: 2.659, 6.551, *p* < 0.001) the likelihood of performing PV toward their children in the future, when compared with parents with a high level of perceived behavioral control over PV. For parents’ who had used violence in the previous three months, a significant effect was also found on their intention to use violent discipline (*p* < 0.05), but this effect was not as strong as that observed for parents’ attitudes toward PV (*OR* = 2.23).

In addition, to better understand whether the effect of TPB constructs on predicting parents’ intention to use PV in the future varied by gender, we performed a multi-group analysis, and the results are shown in Table 4. From Table 4 we can see that gender-disparity is evident between fathers and mothers. For mothers, both their attitudes toward PV (*OR* = 2.41) and perceived behavioral control over PV (*OR* = 4.14) significantly influence their intentions to use PV, but for fathers, only perceived behavioral control over PV (*OR* = 5.28) was associated with their intentions to use PV in the future.

## 4. Discussion

In this study, we found that approximate one in every four parents (23.4%) did not clearly state that they will not use PV in the future. Parents’ attitudes and perceived behavioral control over PV, and parent’s recent use of violent discipline were significantly associated with their behavioral intentions. Further, the study indicates that parents’ perceived behavioral control and attitudes toward PV are more important than perceived social norms regarding use of PV against children.

The evidence suggests that parents’ attitudes toward corporal punishment positively affected their intentions to use PV. Those with attitudes that support PV were more than twice as likely to say they intend to use PV in the future, in comparison to parents who hold positive attitudes toward non-violent discipline. This is consistent with previous studies [23,37]. According to TPB, an individual’s higher intentions to engage in a behavior can lead to a higher probability of practice [28]. Thus, one of the main strategies to reduce parents’ PV behavior toward their children should be to help them change attitudes that tolerate violent discipline. In addition, although PV has negative effects on children’s health, some parents in mainland China consider PV as an effective way to discipline children, and believe that only severe PV should be prevented or intervened by people outside the family [25]. Thus, it is necessary to educate parents about the harmfulness of PV in any form, and the effectiveness of non-violent discipline skills. This might be especially relevant for mothers, as we found evidence of gender disparity in the extent to which attitudes that tolerate violent discipline influences the intention to use it.

This study also found that perceived behavioral control was linked with parents’ intentions to use PV with their children and it might even be more influential than parents’ attitudes toward corporal punishment. Parents with moderate/low perceived behavioral control over PV were four times more likely than other parents to indicate they might use PV in the future. It is possible that, even if parents hold positive attitudes toward non-violent discipline, if they feel overwhelmed by difficulties and cannot effectively use non-violent discipline to educate children, they may resort to violent methods. Thus, teaching parents how to control themselves and especially how to use, and always apply alternative positive parenting skills is important for prevention programs.

In this study, subjective norms were not significantly associated with parents’ intentions to use PV. This finding is similar with other research in other settings, as has been seen in relation to kindergarten teachers’ intentions to report child abuse [16]. This may relate to the relatively limited diversity in parenting culture in China, as many people share similar parenting concepts.

One very interesting observation is that two thirds (67.5%) of parents said they had used some form of physically violent discipline in the previous three months. However, about 76.0% of the sample (including more than half of those who recently used violence) said they will not do so in the future. Only one in four parents said they expect to use violence in the future, or were unsure. This may suggest that many parents actually do not want to discipline their children using violent means. The apparent anomaly could also arise if it is less sensitive to admit that some level of violence has happened in the past than it is to say they willfully choose to do it in the future. Violent discipline is very complex and culturally sensitive; thus, parents’ final behavior may be only partially driven by cognitive, attitudinal and social factors. Further research into parents’ ideals about what they should do, and what they actually practice, may be helpful to clarify these issues.

### Limitations

This study has some limitations in the design and measurement. First, the measurement did not include parents’ perception of the outcomes of physically violent discipline. Other studies have shown that parents’ expectations regarding the harms and benefits from PV are linked with their attitudes toward the use of PV [42]. Second, the measures of subjective norms and perceived behavioral control were self-designed, although we have conducted a reliability analysis and the results were acceptable, further studies are still needed to confirm the reliability and validity of these scales. Third, although we measured parents’ intentions to use PV in the future, the predictive strength of intention to use PV should be explored in future research. Low intention to use PV does not mean that parents will refrain from violence toward their children. Except for measuring parents’ actual behavior over time, to conduct in-depth interviews with non-violent and violent parents would be of great significance to explore factors that hamper parents’ use of non-violent parenting skills to discipline children. Fourth, this study only recruited parents from one county of China, so the results explanation should be limited to the parents who participated in this survey.

## 5. Conclusions

Two components of TPB (parents’ attitudes toward PV and perceived behavioral control over PV) appear to be strongly linked with parents’ intentions to use PV against their children, and the effects may differ according to parents’ gender. Parents’ recent use of violent discipline was also significantly associated with their behavioral intentions. Intervention programs that aim to reduce parents’ PV behavior toward children should focus both on increasing parents’ perceived behavioral control over PV and changing their attitudes toward physically violent practices, especially among mothers and parents who have already used PV to discipline their children.

## Figures and Tables

**Table 1:** Respondents’ demographic characteristics and bi-variable associations with their intentions to use physical violence (PV) in the future.

Variables	Parents Who Intend to or Not Sure to Use PV in the Future n (%)	*χ*^2^ Value
Areas		22.588 ***
Urban (n = 662)	117(17.8)	
Rural (n = 675)	192(28.9)	
Gender		3.029
Father (n = 267)	51(19.3)	
Mother (n = 1070)	258(24.4)	
Parental age, years		5.305 *
>35 (n = 304)	55(18.3)	
≤35 (n = 924)	226(24.7)	
Missing (n = 109)		
Education level		20.249 ***
Senior high school or higher (n = 637)	113(17.9)	
Junior high school or less (n = 682)	191(28.4)	
Missing (n = 18)		
Marital satisfaction		
Satisfied (n = 1112)	239(21.7)	10.667 **
Unsatisfied (n = 210)	66(32.2)	
Missing (n = 15)		
Family economic situation in the local area		3.712
Medium or rich (n = 1168)	258(22.3)	
Poor (n = 147)	43(29.5)	
Missing (n = 22)		
Childhood physical violence victimization by parents		8.276 **
No (n = 389)	70(18.2)	
Yes (n = 938)	237(25.6)	
Missing (n = 10)		
Child gender		0.015
Girl (n = 646)	151(23.5)	
Boy (n = 691)	158(23.2)	
Child age, year		4.772
≥6 (n = 204)	49(24.5)	
4–5 (n = 685)	167(24.7)	
≤3 (n = 395)	75(19.1)	
Missing (n = 53)		
Only-one child family		9.015 **
Yes (n = 161)	22(13.8)	
No (n = 1144)	278(24.6)	
Missing (n = 32)		

Note: * *p* < 0.05, ** *p* < 0.01, *** *p* < 0.001, *PV*: physical violence.

**Table 2:** Descriptive statistics for constructs of the theory of planned behavior (TPB) and bi-variable associations with parents’ intentions to use PV in the future. *n* (%).

Variables	Parents Who Intend to Use or Not Sure to Use PV in the Future	Parents Who do Not Intend to Use PV in the Future	*χ*^2^ Value
Attitudes toward PV			73.255 ***
Against PV (n = 624)	80(12.8)	544(87.2)	
Support PV (n = 692)	227(32.8)	465(67.2)	
Subjective norms regarding PV			42.532 ***
High level (n = 621)	95(15.3)	526(84.7)	
Moderate/low level (n = 687)	210(30.6)	477(69.4)	
Perceived behavioral control over PV			120.771 ***
High level (n = 512)	38(7.4)	474(92.6)	
Moderate/low level (n = 779)	264(33.9)	515(66.1)	
Parental PV behavior to children during the past three months			55.511 ***
No (n = 431)	47(10.9)	384(89.1)	
Yes (n = 891)	262(29.4)	629(70.6)	

Note: *** *p* < 0.001, *PV* physical violence.

**Table 3:** Logistic regression models of factors that predict parents’ intentions to use PV toward their preschool children in the future.

Variable	Univariate *OR* (95% *CI*)	Multivariate *OR* (95% *CI*)		
		Model 1 ^a^	Model 2 ^b^	Model 3 ^c^
Attitudes toward PV				
Against PV	1.00	1.00	1.00	1.00
Support PV	3.32 (2.501–4.407) ***	2.09 (1.522–2.859) ***	1.94 (1.409–2.666) ***	2.23 (1.555–3.203) ***
Subjective norms regarding PV				
High level	1.00	1.00	1.00	1.00
Moderate/low level	2.44 (1.857–3.200) ***	1.26 (0.919–1.719)	1.22 (0.888–1.664)	1.29 (0.902–1.855)
Perceived behavioral control over PV				
High level	1.00	1.00	1.00	1.00
Moderate/low level	6.39 (4.451–9.186) ***	4.81 (3.270–7.086) ***	4.21 (2.833–6.253) ***	4.17 (2.659–6.551) ***
Parental PV behavior to children during the past three months				
No	1.00		1.00	1.00
Yes	3.40 (2.433–4.759) ***		1.72 (1.175–2.523) **	1.62 (1.032–2.556) *

Note: * *p* < 0.05, ** *p* < 0.01, *** *p* < 0.001, PV: physical violence, *CI*: confidence interval, *OR:* odds ratio. ^a^: The multivariate analysis model only included the three constructs of TPB. ^b^: The multivariate analysis model also included parents’ PV behavior during the past three months. ^c^: The multivariate analysis model also adjusted for other variables of respondents’ demographic characteristics showed in Table 1.

**Table 4:** Gender disparity in factors that predict parents’ intentions to use PV toward their preschool children in the future.

Variable	Fathers *OR* (95% *CI*)		Mothers *OR* (95% *CI*)	
	Univariate	Multivariate ^a^	Univariate	Multivariate ^a^
Attitudes toward PV				
Against PV	1.00	1.00	1.00	1.00
Support PV	2.26 (1.197–4.260) *	1.47 (0.562–3.852)	3.61 (2.627–4.963) ***	2.41 (1.621–3.578) ***
Subjective norms regarding PV				
High level	1.00	1.00	1.00	1.00
Moderate/low level	3.29 (1.698–6.374) ***	1.93 (0.719–5.168)	2.26 (1.679–3.054) ***	1.20 (0.812–1.773)
Perceived behavioral control over PV				
High level	1.00	1.00	1.00	1.00
Moderate/low level	7.18 (3.085–16.700) ***	5.28 (1.656–16.815) **	6.16 (4.125–9.204) ***	4.14 (2.526–6.786) ***
Parental PV behavior to children during the past three months				
No	1.00	1.00	1.00	1.00
Yes	3.60 (1.788–7.262) ***	1.77 (0.651–4.823)	3.28 (2.233–4.825) ***	1.64 (0.976–2.756)

Note: *** *p* < 0.001, ** *p* < 0.01, * *p* < 0.05, *PV* physical violence, *CI* confidence interval, *OR* odds ratio. ^a^: The multivariate analysis model also adjusted for other variables of respondents’ demographic characteristics showed in Table 1.

## Supplementary Materials

The following are available online at https://www.mdpi.com/1660-4601/17/5/1787/s1, Table S1: Results of the reliability analysis of the three TPB constructive scales, Table S2: Results of the validity analysis of the three TPB constructive scales, Table S3: Results of the reliability analysis of the PV scale, Table S4: Results of the validity analysis of the PV scale.
