# Supplementary material for: Factors that Influence Chinese Parents’ Intentions to Use Physical Violence to Discipline Their Preschool Children
Source: Int J Environ Res Public Health. 2020 Mar 10;17(5):1787. doi: 10.3390/ijerph17051787 (PMC7084770; doi:10.3390/ijerph17051787)
Supplement: Supplementary file 1 [file ijerph-17-01787-s001.pdf]

Supplementary materials

SPSS was used to evaluate the reliability of **the three TPB constructive scales and PV scale**, indictors including Cronbach's α (> 0.80), Corrected Item-Total Correlation (CITC > 0.40) and Cronbach's Alpha if Item Deleted (ID α < Cronbach's α). AMOS software was used to conduct confirmatory factor analysis (CFA) and Structural Equation Modeling (SEM) to assess the validity of the constructs of the neighborhood collective efficacy scales. Maximum likelihood estimation was used to fit the model. Standardized factor loadings (≥ 0.50), average variance extracted (AVE ≥ 0.50) and composite reliability (CR ≥ 0.70) were calculated to evaluated the construct validity of each scale. Indicators including incremental fit index (IFI > 0.90), goodness-of-fit index (GFI >0.90), adjusted GFI (AGFI > 0.90), comparative fit index (CFI > 0.90), and the root-mean-square error of approximation (RMSEA < 0.08) were used to assess the fit of the model.

Results of the reliability and validity analysis of the three TPB constructive scales

There are 5 items in the attitude scale, 5 items about subjective norms and 6 about perceived behavioral control. The results of the reliability analysis of the three TPB constructive scales were shown in Table S1. The Cronbach's α of the three scales (attitudes, subjective norm, and perceived behavioral control) were 0.81, 0.85, and 0.85, respectively. From Table S1 we can see that, all the CITC value of the three sub-scales were higher than 0.40, and the other items' indicator values were all meet their standards, which means that the reliability of constructs of TPB were acceptable.

Table 1. Results of the reliability analysis of the three TPB constructive scales.

Constructs and items	Cronbach's α	CITC value	IDα
Attitudes	0.81		
Att1		0.66	0.75
Att2		0.65	0.76
Att3		0.64	0.76
Att4		0.58	0.78
Att5		0.46	0.81
Subjective Norms	0.85		
SN1		0.61	0.84
SN2		0.70	0.81
SN3		0.64	0.83
SN4		0.71	0.81
SN5		0.67	0.82
Perceived behavioral control	0.85		
PBC1		0.73	0.80
PBC2		0.55	0.84
PBC3		0.53	0.84
PBC4		0.71	0.81
PBC5		0.70	0.81
PBC6		0.58	0.83

The results of the validity analysis (CFA) of the three TPB constructive scales were shown in Table S2. From this table we can see that except of the AVE of attitudes scale was a bit lower than 0.50, the AGFI of subjective norms scale was a bit lower thatn 0.90, and the RMSEA of subjective norms scale was higher than 0.08, other values of item loading, AVE, and CR were all meet their standards, which means that the reliability of constructs of TPB were acceptable.

Table 2. Results of the validity analysis of the three TPB constructive scales.

**Constructs and items**	**AVE**	**CR**	**Item loading**	***P Value***
Attitudes	0.47	0.81		
Att1			0.78	fixed
Att2			0.77	<0.001
Att3			0.72	<0.001
Att4			0.63	<0.001
Att5			0.48	<0.001
Fit indices of the measurement models: Relative Chi-Square = 47.730, P<0.001; IFI (>0.90) =0.979; GFI (>0.90) =0.986; AGFI (>0.90) =0.957; CFI (>0.90) =0.979; RMSEA (< 0.08) =0.080 (Standardized estimates)				
Subjective Norms	0.54	0.85		
SN1			0.66	fixed
SN2			0.75	<0.001
SN3			0.70	<0.001
SN4			0.80	<0.001
SN5			0.77	<0.001
Fit indices of the measurement models: Relative Chi-Square = 153.378, P<0.001; IFI (>0.90) =0.947; GFI (>0.90) =0.952; AGFI (>0.90) =0.857; CFI (>0.90) =0.947; RMSEA (< 0.08) =0.149 (Standardized estimates)				
Perceived behavioral control	0.50	0.85		
PBC1			0.81	fixed
PBC2			0.59	<0.001
PBC3			0.57	<0.001
PBC4			0.78	<0.001
PBC5			0.78	<0.001
PBC6			0.64	<0.001
Fit indices of the measurement models: Relative Chi-Square = 72.942, *P*<0.001; IFI (>0.90) =0.979; GFI (>0.90) =0.981; AGFI (>0.90) =0.956; CFI (>0.90) =0.979; RMSEA (< 0.08) =0.073 (Standardized estimates)				

Results of the reliability and validity analysis of the PV scale

There are 8 items in the PV scale. The results of the reliability analysis of the PV scale was shown in Table S3. The Cronbach's α of scale was 0.85. From Table S3 we can see that, all the CITC value of the PV scale were higher than 0.40, and the other items' indicator values were all meet their standards, which means that the reliability of PV scale was acceptable.

Table 3. Results of the reliability analysis of the PV scale.

Items	CITC value	IDα
Item 1	0.66	0.82
Item 2	0.68	0.82
Item 3	0.62	0.83
Item 4	0.66	0.82
Item 5	0.60	0.83
Item 6	0.58	0.83
Item 7	0.52	0.84
Item 8	0.49	0.84

The results of the validity analysis (CFA) of the PV scale was shown in Table S4. From this table we can see that the AVE of PV scale was a bit lower than 0.50, the IFI, AGFI, and CFI of PV scale was a bit lower than 0.90, and the RMSEA of subjective norms scale was higher than 0.08. Other values of item loading, and CR were all meet their standards. The reliability of PV scale was acceptable to some extent.

Table 4. Results of the validity analysis of the PV scale.

Items	AVE	CR	Item loading	*P Value*
PV scale	0.43	0.86		
Item 1			0.71	fixed
Item 2			0.73	<0.001
Item 3			0.67	<0.001
Item 4			0.72	<0.001
Item 5			0.66	<0.001
Item 6			0.64	<0.001
Item 7			0.58	<0.001
Item 8			0.54	<0.001
Fit indices of the measurement models: Relative Chi-Square = 440.481, *P*<0.001; IFI (>0.90) =0.892; GFI (>0.90) =0.914; AGFI (>0.90) =0.845; CFI (>0.90) =0.892; RMSEA (< 0.08) =0.125 (Standardized estimates)				
